# Complete Genome Sequences of Four European Subtype Strains of Tick-Borne Encephalitis Virus from Eastern Siberia, Russia

**DOI:** 10.1128/genomeA.00609-15

**Published:** 2015-06-18

**Authors:** Renat V. Adelshin, Olga V. Melnikova, Ludmila S. Karan, Evgeny I. Andaev, Sergey V. Balakhonov

**Affiliations:** a Irkutsk Anti-Plague Research Institute of Siberia and Far East, Rospotrebnadzor, Irkutsk, Russia; b Irkutsk State University, Irkutsk, Russia; c Central Institute of Epidemiology, Rospotrebnadzor, Moscow, Russia

## Abstract

Three tick-borne encephalitis virus (TBEV) strains were isolated from *Ixodes persulcatus* ticks, and one was isolated from a shrew in the territory of eastern Siberia (Russia). The level of sequence identity compared to Neudoerfl (the European prototype strain) is 97.2 to 97.3%.

## GENOME ANNOUNCEMENT

There are three main subtypes of tick-borne encephalitis virus (TBEV): 1, Far-Eastern; 2, European (Western); and 3, Siberian (1). Each subtype dominates its respective area. However, there are many cases when the TBEV subtypes have been detected in unusual areas, such as the Siberian subtype in Europe (2, 3), the Far-Eastern subtype in Crimea (4, 5) and the European part of Russia (6), and the European subtype in South Korea (7). The Siberian TBEV subtype absolutely dominates in the eastern Siberia nowadays, the Far-Eastern subtype is now found not so often as 30 to 40 years ago, and the European subtype occurs in isolated cases (8).

The TBEV strains of the European subtype reported to date in eastern Siberia have been isolated from small mammals and from a blood sample of the only recorded infected patient (8). We present here the complete genome sequences of four European subtype strains of TBEV isolated from *Ixodes persulcatus* ticks, collected by flagging in TBE natural foci throughout Irkutsk city suburbs, and from the brain of a shrew (*Sorex* sp.) captured in one of these foci. The strains were named IrkutskBR_99-08, IrkutskBR_1434-09, IrkutskBR_1456-09, and Sorex 18-10.

The strain IrkutskBR_99-08 was isolated by infecting porcine embryo kidney cells with an enzyme-linked immunosorbent assay (ELISA)-negative male tick suspension. The strains IrkutskBR_1434-09 and IrkutskBR_1456-09 were isolated by intracerebral inoculation of newborn nonlinear laboratory mice with a poor ELISA-positive female tick suspension. The strain Sorex 18-10 was isolated by inoculating newborn nonlinear laboratory mice with 10% saline brain suspension. Each strain is highly neurotropic for laboratory mice whether inoculated through the intracerebral or peripheral route.

Total RNA was extracted from brain tissue (10% suspension in saline) using the RiboPrep kit (ILS Ltd., Russia). Reverse transcription was performed by means of the Reverta-L 100 kit containing random hexanucleotides (Central Research Institute of Epidemiology, Moscow, Russia). Amplification of cDNA fragments was performed with a PCR kit (Sintol, Moscow, Russia) and a set of primers (see the GenBank submissions). The PCR products were sequenced with the ABI Prism BigDye Terminator version 1.1 cycle sequencing kit on the Genetic Analyzer 3500xl (Applied Biosystems). The sequences (10,471 bp) were aligned in the BioEdit version 7.0.5.3 (9). Phylogenetic analysis was performed using the MEGA5 program (10) using the maximum-likelihood method.

Each nucleotide sequence was compared to that of the European prototype strain Neudoerfl (U27495), and the following identities were shown: 97.2% for IrkutskBR_99-08 and 97.3% for IrkutskBR_1434-09, IrkutskBR_1456-09, and Sorex 18-10.

To summarize, we have provided the first complete genome sequences of three European subtype strains of TBEV isolated from taiga ticks (*I. persulcatus*) and from a shrew (*Sorex* sp.) in eastern Siberia.

### Nucleotide sequence accession numbers.

The complete sequences have been deposited in GenBank under the accession numbers KP331441 (IrkutskBR_99-08), KP331442 (IrkutskBR_1434-09), KP331443 (IrkutskBR_1456-09), and KP938507 (Sorex 18-10).
